# An evaluation of the risk of airborne transmission of COVID‐19 on an inter‐city train carriage

**DOI:** 10.1111/ina.13121

**Published:** 2022-10-24

**Authors:** Huw Woodward, Rick J. B. de Kreij, Emily S. Kruger, Shiwei Fan, Arvind Tiwari, Sarkawt Hama, Simon Noel, Megan S. Davies Wykes, Prashant Kumar, Paul F. Linden

**Affiliations:** ^1^ Centre for Environmental Policy Imperial College London London UK; ^2^ Department of Engineering University of Cambridge Cambridge UK; ^3^ Department of Applied Mathematics and Theoretical Physics, Centre for Mathematical Sciences University of Cambridge Cambridge UK; ^4^ Department of Chemistry University of Cambridge Cambridge UK; ^5^ Global Centre for Clean Air Research (GCARE), Department of Civil & Environmental Engineering University of Surrey Guildford UK; ^6^ QinetiQ Ltd. Farnborough UK

**Keywords:** airborne transmission, CO_2_, public transport, SARS‐CoV‐2, ventilation

## Abstract

Experiments were conducted in an UK inter‐city train carriage with the aim of evaluating the risk of infection to the SARS‐CoV‐2 virus via airborne transmission. The experiments included in‐service CO_2_ measurements and the measurement of salt aerosol concentrations released within the carriage. Computational fluid dynamics simulations of the carriage airflow were also used to visualise the airflow patterns, and the efficacy of the HVAC filter material was tested in a laboratory. Assuming an infectious person is present, the risk of infection for a 1‐h train journey was estimated to be 6 times lower than for a full day in a well‐ventilated office, or 10–12 times lower than a full day in a poorly ventilated office. While the absolute risk for a typical journey is likely low, in the case where a particularly infectious individual is on‐board, there is the potential for a number of secondary infections to occur during a 1‐h journey. Every effort should therefore be made to minimize the risk of airborne infection within these carriages. Recommendations are also given for the use of CO_2_ sensors for the evaluation of the risk of airborne transmission on train carriages.

**Practical implications**

- This work contributes to the small amount of experimental data currently available in the literature aimed at characterising the risk of airborne infection on inter‐city trains.
- Our analysis shows that the train carriage can be approximated as well‐mixed along its height and width, but not along its length, along which there can be a significant variation in CO_2_ concentrations.
- The HVAC filter material currently used leads to an enhancement in the removal of airborne droplets of only 5%–8% above that provided by the provision of fresh air.
- The risk of infection is likely to be low for one off journeys, however, can be significant in the presence of a particularly infectious individual.

## 1 INTRODUCTION

Journeys taken on inter‐city trains were identified as potentially high risk for COVID‐19 infection due to the longer journey times, difficulty in increasing fresh air supply rates on mechanically ventilated carriages, and the challenge of enforcing physical distancing within a small space. Train operators can enforce mask‐wearing, physical distancing when possible, and apply antimicrobial products to surfaces to mitigate transmission via larger droplets and fomites, respectively. However, mitigating the risk of airborne transmission (i.e., transmission via small infectious particles) is often more difficult.^1^ While wearing a mask does reduce the likelihood of airborne transmission,^2^ there is a wide range in the effectiveness of masks and the risk is rarely eliminated completely, even for the highest protection level masks such as FFP3, due to imperfect fit or adherence.^3^

Until recently, while studies of airborne transmission existed for other vehicle types such as aircraft,^4^, ^5^, ^6^, ^7^ equivalent studies for train journeys have been few and far between. Most studies of airflow patterns on trains focused on the thermal comfort of passengers.^8^, ^9^ Since the beginning of the COVID‐19 pandemic, there has been a shift in focus driven by a motivation to quantify and understand how to mitigate the risk of transmission of airborne diseases. An epidemiological study of infections on high‐speed trains in China by Hu et al.^10^ concluded that there was a significant risk of infection and recommended that measures such as the use of personal protective equipment and maximising distance between passengers should be used. A modelling study of the infection risk on various train types in Germany came to similar conclusions,^11^ demonstrating that the risk of infection can be reduced by as much as two orders of magnitude by enforcing the wearing of FFP2 masks. Shinohara et al.^12^ used CO_2_ sensors to evaluate the risk of infection on naturally ventilated trains and found that by opening all windows the risk could be reduced by over 90%. Ahmadzadeh and Shams^13^ used computational fluid dynamics simulations to demonstrate that increasing the rate of fresh air supply on a mechanically ventilated train also leads to a significant reduction in the risk of airborne transmission of COVID‐19. Increasing the fresh air supplied to any indoor space is known to be an effective strategy for reducing airborne transmission.^1^ However, mechanically ventilated inter‐city train carriages are optimised for thermal comfort and energy efficiency^14^ and are not designed for flexibility in terms of varying airflow rates.

This paper brings together results from various experiments and computational fluid dynamics (CFD) simulations to provide an understanding of the risk of airborne transmission of COVID‐19 within an inter‐city train carriage operating within the UK. The experiments conducted on the carriage include in‐service CO_2_ and temperature measurements taken during the months of November 2020 and 2021 over a total course of 3 days, and measurements of the concentrations of salt aerosol droplets released from a nebuliser within the saloon. Laboratory tests of the efficacy of the HVAC filter material in removing sub‐10 micron particles were also performed. The risk of airborne transmission was estimated using carbon dioxide (CO_2_) concentrations as a proxy for re‐breathed air, as done in Burridge et al.^15^ The use of measured CO_2_ concentrations to estimate the risk of airborne transmission was also evaluated using the findings from the various experiments and CFD.

It is important to note that within this paper we focus on the risk of transmission due to airborne droplets only. We define “airborne” droplets as aerosols which are sufficiently small to remain in the air for more than a few seconds, and for which dilution through increased ventilation is an effective mitigating strategy. Large droplets which fall to the ground within seconds are not considered within the risk calculations as exposure to these droplets cannot be mitigated through increased ventilation. We do not define a specific size threshold between “small” and “large” droplets. We do, however, consider the difference in dispersion for two droplet size ranges (≤2.5 μm and 2.5–10 μm) within the carriage through the aerosol droplet measurements.

In Section 2, we describe the methods used for the various experiments and simulations, and we present and discuss the results in Section 3. We then provide our conclusions in Section 4.

## 2 METHODOLOGY

### 2.1 Carriage layout and ventilation

The carriage is made up of a saloon and two vestibules on either end, which are connected by automatic sliding doors. Each vestibule has an exterior door on either side, as well as a door which leads to the adjacent carriage via a gangway connection.

The saloon has a length of 19.85 m, an average width of 2.69 m and a height of 2.02 m, resulting in a volume of 108 m^3^. The vestibules have a similar width and height as the saloon, but their length varies between the 2.00 m and 2.50 m, which results in a volume around 16 m^3^. In terms of capacity, the saloon offers 88 chairs, with 8 tables of 4 chairs and 28 separate pairs. The crush capacity of the carriage is 160, with 72 standing.

**FIGURE 1:**
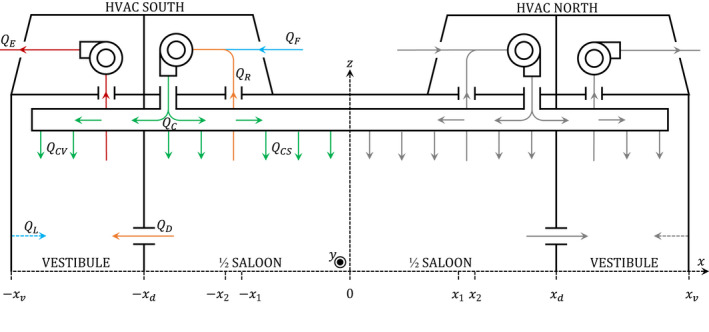
Simplified ventilation diagram of the symmetric carriage. The carriage has two HVAC systems, sharing the same outlet over the length of the carriage. Each HVAC system consists of an evaporator and exhaust fan. The flow rates corresponding to half of the carriage are indicated with the colored arrows: fresh air (blue), circulated air (green), recirculated air (orange), extracted air (red). The HVAC filters are located at the recirculated air inlet ($Q_{R}$).

The carriage is mechanically ventilated by two HVAC systems, which are located on the roof at either end of the carriage, as shown in the ventilation schematic in Figure 1. Each HVAC system introduces fresh air to the carriage, recirculates air from the saloon, and extracts air from the vestibule.

The HVAC is driven by an evaporator and exhaust fan. The evaporator fan draws in recirculated air from the saloon and fresh air from outside. This evaporator fan also pumps air into the HVAC outlet, where it is distributed into the saloon and into the vestibule.

**FIGURE 2:**
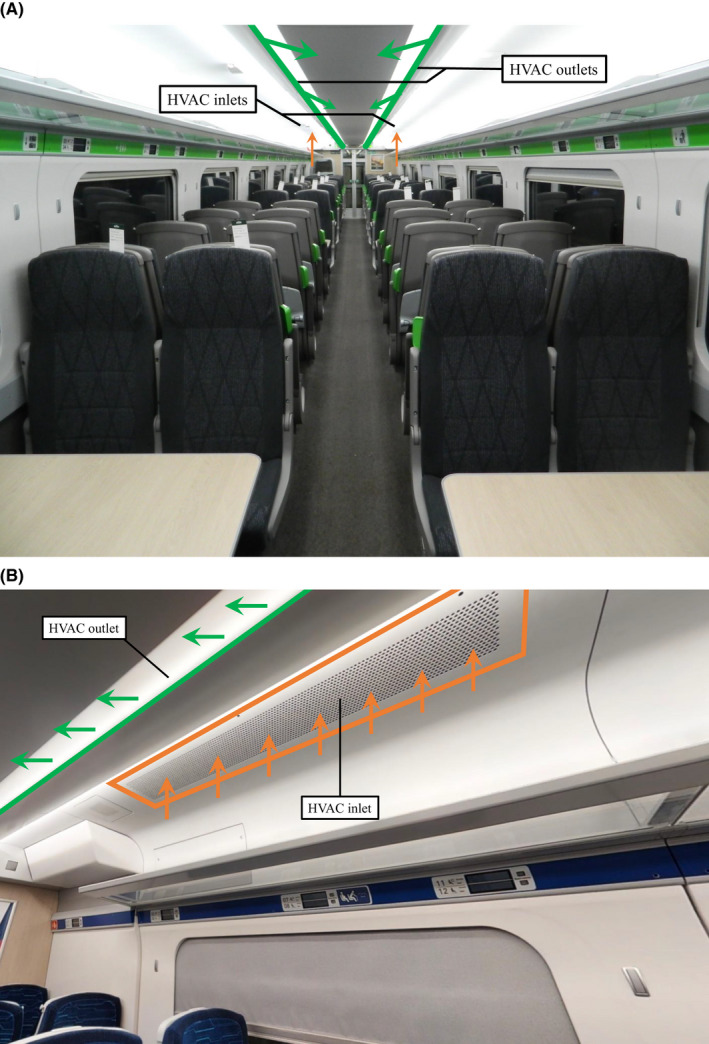
Interior of the carriage saloon, including the HVAC inlet (orange) and HVAC outlet (green). The arrows indicate the flow direction at the in‐ and outlets.

Air enters the saloon through the HVAC outlets, which consist of two ceiling slits which extend the entire length of the saloon, as shown in Figure 2. These slits are orientated toward the centre of the saloon at an angle. These HVAC outlets are shared between the two HVAC systems.

The extraction of air from the saloon is also driven by the evaporator fan through the HVAC inlets and consists of two rectangular ceiling grids per HVAC system, as shown in Figure 2. These HVAC inlets are positioned on each side near the end of the saloon, beginning at a distance of 6.9 m and ending at 8.0 m from the centre.

The exhaust fan is mounted near the end of the carriage and drives the extraction of air from the carriage. Air is only directly extracted from the vestibule and not from the saloon. Air is extracted from the saloon via the recirculated air distributed into the vestibule, $Q_{\mathrm{CV}}$, and airflow through the doorway connecting the vestibule and the saloon, $Q_{D}$. In turn, this airflow from the saloon to the vestibule drives the intake of fresh air into the carriage, $Q_{F}$.

The design flow rates of the carriage are listed in Table 1. The Air Change per Hour (ACH) for the design flow rates varies between 11 and 15 ACH. The HVAC system operated on automatic mode for the duration of the experiments and therefore the exact flow rates were not known. On automatic mode the HVAC unit adjusts the airflow rates in response to the in‐carriage and external temperature but is not equipped with CO_2_ monitors and therefore does not respond to elevated CO_2_ concentrations.

**TABLE 1:** Design flow rates for a single HVAC system (half of carriage) in m^3^ min^−1^

Flow rate	$Q_{F}$	$Q_{C}$	$Q_{\mathrm{CS}}$	$Q_{\mathrm{CV}}$	$Q_{R}$	$Q_{D}$	$Q_{L}$	$Q_{E}$
min	11.3	30	–	–	15	–	0	11.3
max	15	45	40	5	30	10	0	15

### 2.2 CFD simulations

Unsteady Reynolds‐Averaged Navier–Stokes (URANS) Computational Fluid Dynamics simulations were run in order to understand the airflow within the carriage saloon. The CFD package Star‐CCM+ was used with a polyhedral mesh comprising approximately 22 million cells and an implicit unsteady formulation with a k−ε realisable turbulence model. Details of the setup of the CFD simulations are given in Appendix S1.

Here, we primarily focus on figures of the airflow within an empty saloon in order to gain an understanding of the bulk flow directions and potential areas of recirculating or stagnant air. However, two simulations of a carriage occupied by 6 people were also considered and are included in Appendix S1.

### 2.3 Aerosol particle measurements

When attempting to understand the dispersion behavior of exhaled droplets, it is important to consider the size distribution of these droplets. Larger droplets tend to fall to the ground at a quicker rate than smaller droplets and are not transported as far or for as long a time.

There is a considerable variation in the exhaled droplet size distribution between subjects and depending on activity. The vast majority of droplets exhaled while breathing at rest tend to be <2.5 μm in diameter. When talking, larger droplets are also produced, within the 2.5–10 μm range (e.g., see Morawska et al.^16^), leading to the vast majority of the droplet mass being attributed to this range. Droplets that are >10 μm are also produced however the number is typically smaller. Further, in the occurrence that the subject is wearing a mask these larger droplets are likely to be captured by the mask.

**FIGURE 3:**
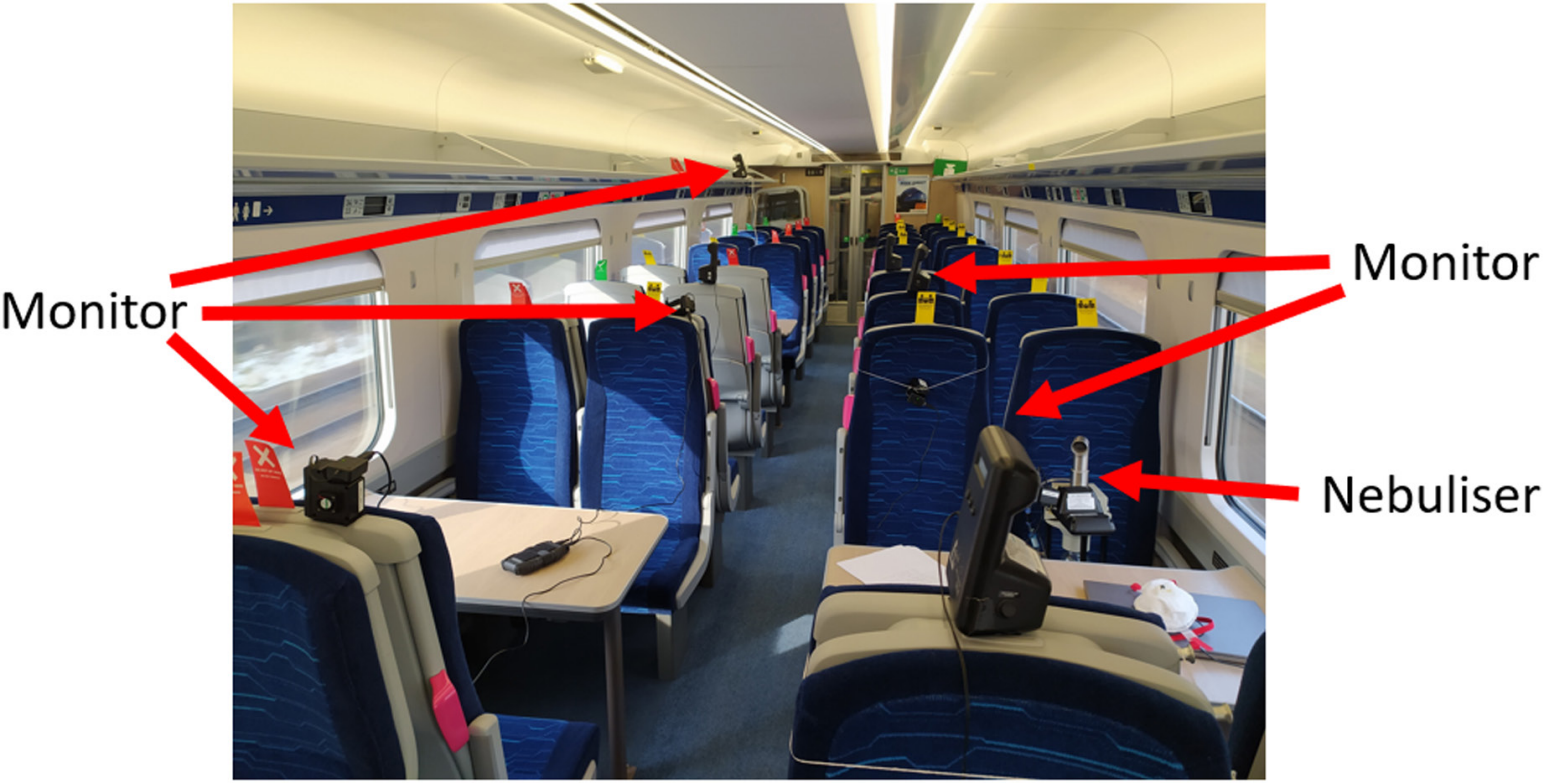
Typical setup of nebuliser experiment.

Measurements of aerosol particle concentrations released by a nebuliser were carried out within the carriage. These experiments were conducted on an empty train during driver training exercises while the train traveled back and forth between London and Doncaster. The experiments involved the release of salt aerosol droplets (1% salt by weight) within the carriage and the measurement of aerosol concentrations at different locations. Eighteen portable aerosol monitors were used including 10 Dylos 1700,^17^ 7 OPC‐N3 Alphasense^18^ and one research grade instrument GRIMM Model EDM 107. To ensure the quality of the data, we carried out measurements co‐located with a research grade instrument for a period of 8 h (Appendix S1: Section S3). The Pearson correlation coefficients (r) between the research grade instrument and portable aerosol monitors were greater than 0.85, and 0.83 for PM_2.5_, and PM_10_, respectively (Figure S6, S7). The sensors were either placed on tables, on the back of passenger seats or on the luggage rack near the ceiling and at various distances along the length of the carriage. The size distribution of the droplets released by the nebuliser is shown by the blue line in Figure S5. The released droplet size ranges from around 0.01–10 μm. A flow rate of 6 L/min was used, which is within the range of the human breathing rate while resting of 5–7 L/min. Battery‐operated aerosol monitors were mounted at different locations and heights within the saloon and the vestibule in each experiment. Two size fractions of the aerosol were measured; ${\mathrm{PM}}_{2.5-10}$ consisting of all aerosol particles with an aerodynamic diameter, d, 2.5 μm ≤ *d* ≤ 10 μm, and ${\mathrm{PM}}_{2.5}$ consisting of all aerosol particles with an aerodynamic diameter, d≤2.5 μm. Figure 3 shows a typical setup for the experiment.

The experiments were conducted for two different release locations. In Woodward et al.,^14^ significantly different airflow patterns and CO_2_ decay curves were observed between the end of the saloon, near the HVAC inlets, and the middle of the saloon. The aerosol droplets were therefore released both at an “end” location (third row of seats from the end) and a “middle” location, near the middle of the saloon. Five runs were conducted at the “end” location and six runs at the “middle” location. Each experiment lasted between 30 and 50 min by which time a steady‐state concentration was reached for all locations other than for the monitor placed directly next to the source itself.

An important consideration when measuring the transport of droplets through the air is evaporation. The relative humidity (RH) within the carriage during these experiments was low; ranging between 12% and 20%. The temperature remained around 21°C. For these conditions, evaporation will occur rapidly for larger droplets (<0.2 s), leading to a significant reduction in size.^19^ It is also worth noting that for an in‐service carriage, the RH is likely to be higher due to the presence of a greater number of people. During the in‐service measurements discussed in Section 2.5, the RH ranged between 30% and 50%, while the temperature was reasonably steady at near 21°C. Sufficient concentrations of the larger droplet size range were measured to provide insight into the dispersion of this larger range under these particular conditions, however to fully understand the behavior of both size ranges experiments should be conducted over the full operational range of RH and temperatures.

### 2.4 HVAC filter laboratory tests

A novel filter testing rig (Figure S3) was used in a laboratory to measure the filtration performance of the carriage filter material in the sub‐10 micron size range at a flow rate of 10 L min^−1^,^2^ which is lower than the flow rate of the carriage HVAC system. Detailed assessment of size‐segregated aerosols in the sub‐10 micron range revealed that the nebuliser could produce constant and uniform particles in the range 0.01–10 μm for testing both new and used samples. A fast response differential mobility spectrometer (Cambustion Instruments DMS500, Cambustion) was used in conjunction with the state‐of‐the‐art solenoid switching system to pseudo‐simultaneously measure particle number distribution before and after the sample filter.

For evaluating the performance of sample filters, the filtration efficiency was estimated using

(1) $$\text{Filtration Efficiency}=\frac{C_{\text{before}}-C_{\text{after}}}{C_{\text{before}}}$$

Filtration efficiency is a common metric for measuring particle capture efficiency. The pressure difference (dP) was also obtained across the filter using $\mathrm{dP}=P_{\text{before}}-P_{\text{after}}$.

Two samples, a new and a used sample (Figure S4), were cut into 5 cm by 5 cm square size and used for analysis in the rig.

### 2.5 In‐service CO_2_ measurements

**FIGURE 4:**
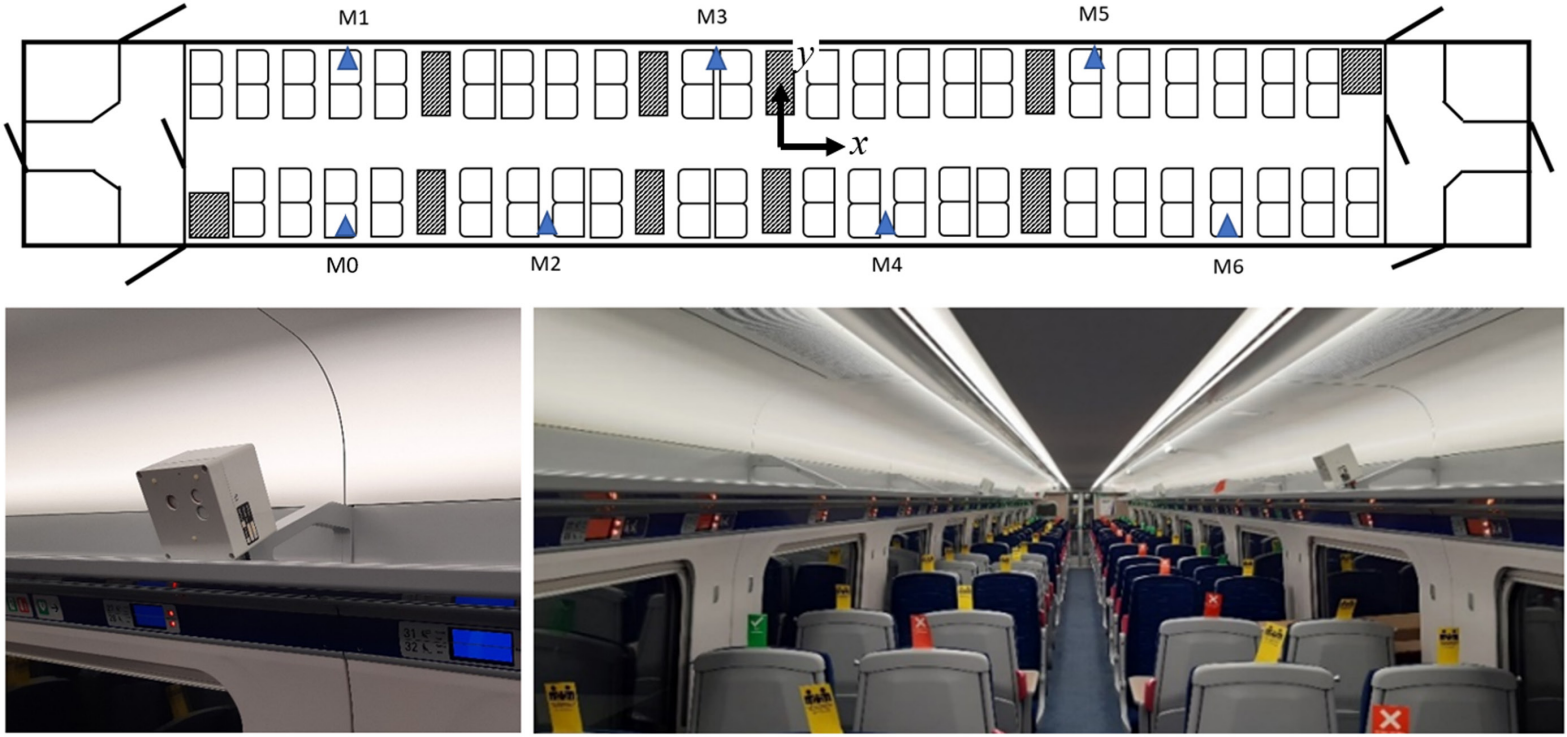
Location of CO_2_ sensors for in‐service measurements. Blue triangles indicate CO_2_ sensors.

It was arranged for CO_2_ sensors (K33‐LP T, SenseAir AB) to be placed in the carriage while in service travelling between London and Hull. The sensors are based on the non‐dispersive infrared (NDIR) principle, which measures the absorptance of infrared light that is proportional to the CO_2_ concentration. The sensors were calibrated with a reference analyser (G2201‐i, Picarro Inc). The percentage error of reading was within 3% in the range of 0–3000 ppm, with a typical accuracy within 50 ppm. Measurements were carried out twice, once in November 2020 and once in November 2021. Six sensors were placed on the luggage shelves at various locations, (M1‐6), along the length of the carriage as seen in Figure 4. In addition to these sensors, during the November 2021 measurements an additional 5 CO_2_ sensors were placed at location M0 and M1 at 3 different heights. Furthermore, five thermistors were also arranged vertically between the floor and ceiling at location M1. The occupancy of the carriage was manually counted and for the main leg of the journey, between London and Grantham, the locations of the passengers were also noted. This leg of the journey takes between 60 and 70 minutes. Both experiments took place during periods of high prevalence of the virus in the community, with the 2020 experiment taking place shortly before the start of the second UK lockdown. The number of passengers on the service were therefore low, with a maximum occupancy of 35. The 2020 experiment was also cut short due to the lockdown and therefore only 2 days of data was gathered, with an additional day of data gathered in 2021.

A well‐mixed model, outlined by de Kreij et al.^20^ and applied previously to other scenarios, for example classrooms,^21^, ^22^ is used to estimate the CO_2_ concentrations within the saloon. The model equation is given as follows

(2) $$C\left(t\right)=C_{\mathrm{ex}}+\frac{G}{Q}+\left(C_{\text{in}}-C_{\mathrm{ex}}-\frac{G}{Q}\right)e^{\left(-\frac{Q}{V}t\right)}$$

where $C\left(t\right)$ is the concentration of CO_2_ in the saloon at time t, $C_{\mathrm{ex}}$ is the external CO_2_ concentration, assumed to be 400 ppm, and $C_{\text{in}}$ is the initial concentration, taken as the mean measured value at the beginning of each journey. G is the CO_2_ generation rate within the saloon, Q is the rate of fresh air supply, and V is the volume.

### 2.6 Estimating airborne infection risk from CO_2_ measurements

We can estimate the risk of airborne transmission using CO_2_ concentrations as a proxy for re‐breathed air, for example, see Burridge et al.^15^ and Rudnick & Milton.^23^ Then, the probability of an individual being infected is given by,

(3) $$P=1-\exp\left(-\lambda \right)$$

where λ is the infectivity rate and is estimated using,

(4) $$\lambda =\frac{I}{n}\mathrm{qfT}$$

Here, n is the number of occupants of which I are infected, and q is the quanta generation rate, defined as the number of infectious particles required to infect a susceptible person.^24^ The fraction of re‐breathed air, f, is given by $f=\left(C-C_{\mathrm{ex}}\right)/C_{a}$ where *C* is the measured CO_2_ concentration in the carriage, $C_{\mathrm{ex}}$ is the outdoor CO_2_ concentration, and $C_{a}$ is the CO_2_ concentration of exhaled breath ($C_{a}$ = 3.8% = 38 000 ppm^21^). Finally, T is the time of exposure.

The total number of secondary infections $S_{I}$ that will occur can then be estimated from Equation 5:

(5) $$S_{I}=\left(n-1\right)\;P$$

The infection probability of Equation 3 and secondary infections of Equation 5 assume I infected occupants within the space. Therefore, the probability of I infected persons being present in the first place is not accounted for. The likelihood of I infected persons being present depends on the number of occupants, n, and the prevalence of the virus in the community, ζ. The probability of infection, P and, the number of secondary infections, S, when the infection rate is ζ, is given by,

(6) $$P=\sum_{I=1}^{n-1}\left(\left(\begin{array}{c} n-1\\ I \end{array}\right)\zeta^{I}{\left(1-\zeta \right)}^{n-1-I}\left(1-e^{-\lambda \left(I\right)}\right)\right)$$

(7) $$S=\sum_{I=1}^{n-1}\left(\left(n-I\right)\left(\begin{array}{c} n-1\\ I \end{array}\right)\zeta^{I}{\left(1-\zeta \right)}^{n-1-I}\left(1-e^{-\lambda \left(I\right)}\right)\right)$$

### 2.7 Quanta generation rate

In order to estimate the risk of infection, an estimate of the quanta value, q, is required. The viral load released by infected persons is known to vary significantly from one person to the next. It varies between individuals and also depends on the activity level of the individual, for example heavy breathing due to exercise, or talking loudly, results in a greater release of infected aerosol as compared to when at rest.^25^ The quanta value also depends on the variant of the virus with which the person is infected.^26^ For example, the Omicron variant, which at the time of writing is the dominant strain in the UK, is significantly more infectious than other variants, with reproduction number estimates four times or greater than that of the original variant.^26^, ^27^ The infectious load required to infect a person will also depend on their vaccination status.

Due to the highly dynamic nature of the pandemic, any estimates of absolute infection risk are likely to be speculative, and to become outdated very quickly. Further, estimates of quanta values representative of the current situation in the UK are not available. We therefore use low and high estimates of the quanta value derived from studies conducted towards the beginning of the pandemic in 2020, during which time the original variant was dominant. A value of 1 h^−1^ is taken as the low estimate and 100 h^−1^ as the high estimate, taken from the work of Buonanno et al.^25^ who based their estimates on the viral load in the mouth and rate of droplet emission for various activities. The risk values estimated should therefore not be considered as representative of the current risk of rail travel in the UK. However, by taking these low and high estimates, we hope to demonstrate a range of risks that was present near the beginning of the pandemic.

It should also be noted that, in the most part, passengers on a train carriage are passive and therefore a lower quanta value is most likely to represent the average case.

## 3 RESULTS AND DISCUSSION

### 3.1 Airflow within the carriage saloon

**FIGURE 5:**
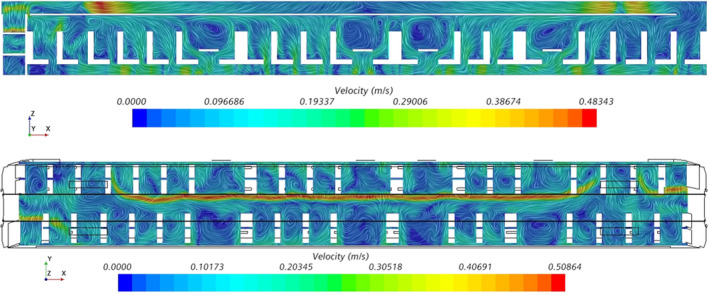
Plane views of the instantaneous velocity within an empty carriage for X‐Z plane (top) and X‐Y plane at Z = 1.0 m (bottom).

**FIGURE 6:**
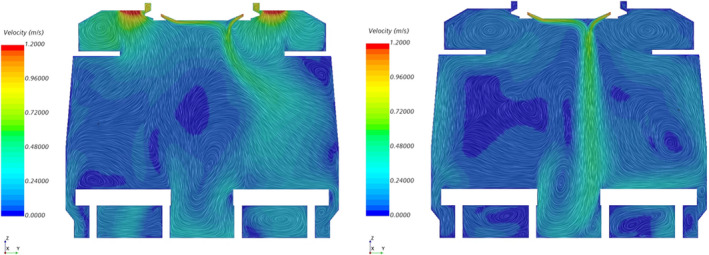
Y‐Z plane views of the instantaneous velocity within an empty carriage immediately below the HVAC inlet vents (left) and near centre of the carriage (right).

Figure 5 shows the instantaneous velocity within the carriage after 30 min of simulation time. The jet of air from the HVAC outlet is clearly visible and has a clear bias toward one side of the carriage. The jet is not visible at a height of 1 m near each end of the saloon due to the interaction of the jet with the suction of the HVAC inlets, as seen in the left‐hand image of Figure 6, which shows the Y‐Z plane at the HVAC inlet location. There is a clear difference in the flow patterns between this location and towards the centre of the saloon, shown on the right‐hand side of Figure 6, where a strong jet is seen to extend the entire height of the saloon. There may be a degree of short‐circuiting in terms of the fresh air supply into the saloon as some of the air from the HVAC outlets is drawn back directly into the HVAC inlet, however this effect is likely to be minimal as it occurs only directly next to the outlet vents.

The difference in flow patterns between the end and the middle of the saloon was also observed during flow visualisation experiments conducted on the carriage and described in Woodward et al.^14^ However, the jet observed during these experiments was highly unstable and tended to fluctuate between one side of the carriage and the other. The jet seen in the CFD figures seems to be stronger and more stable than the experiments showed. This stability may be due to the use of URANS which does not capture the full turbulence of the flow. Despite this, both the flow visualisation experiments and CFD simulations suggest that, at least away from the HVAC inlets, the jet is effective at mixing the air vertically.

Areas of recirculating air are clearly visible between the rows of chairs in Figure 5, with particularly strong vortices visible above the tables. These areas of recirculating air could lead to an accumulation of “older” air (i.e., not fresh air from outside) and therefore the potential for the accumulation of viral‐laden droplets. However, this was not reflected in simulations which included CO_2_ released within the carriage; higher concentrations of CO_2_ were not observed in these areas (see Figure S2). Rather, CO_2_ simulations revealed the CO_2_ to be well‐mixed along both the width and height of the carriage.

**FIGURE 7:**
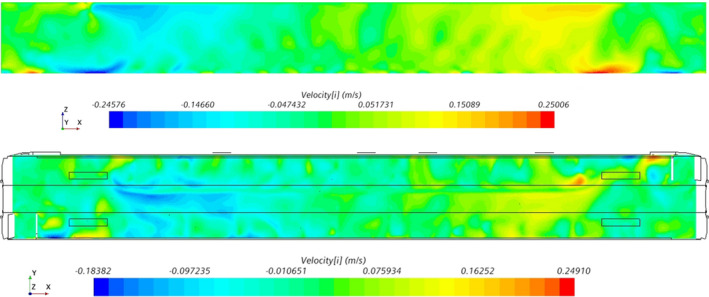
Plane views of the X component of velocity for the X‐Z plane (top) along the aisle and X‐Y plane at Z = 1.4 m (bottom).

Figure 7 shows the along‐carriage component of the airflow velocity within the saloon. The bulk airflow direction within the saloon is toward the nearest HVAC inlet. As the two HVAC inlets are situated at either end of the saloon, there is a symmetry to the bulk airflow direction, with a near‐zero along‐carriage velocity at the centre of the saloon.

An aspect which is missing from our analysis is the effect of human‐induced dispersion within the carriage. Evidence within the literature suggests that the effect of human movement on the airflow, and resulting contaminant dispersion, within indoor environments is not negligible and can be significant.^28^, ^29^, ^30^ Human movement along the aisle of the carriage could lead to enhanced mixing along the length of the carriage. The motion of walking has also been shown to induce vertical mixing.^29^ In this case, any enhanced vertical mixing is likely to be less important due to the fact that the saloon is already well‐mixed vertically. However, any increased mixing along the length of the carriage will alter the exposure of passengers to airborne particles, and therefore their risk of infection. The importance of this effect will depend on a number of variables, including the carriage occupancy, the number and frequency of passengers who walk along the aisle and the ventilation rates during these occurrences.

For an inter‐city train carriage, the frequency with which people move up and down the carriage is likely to be lower than for regional trains due to the longer periods between stops. Further, the effect of human movement on the airflow becomes less important with higher ACH,^29^ which is high relative to most other indoor environments. Despite this, further work is required in order to establish the importance of this effect within the carriage.

A second human‐induced aspect which affects the airflow within the carriage is the effect of the body plume of passengers. In Woodward et al.^14^ the body plume of passengers was clearly visible in flow visualisations performed within the carriage. While the body plume does affect the flow within the carriage, for the cases considered so far its effect seems mainly to further enhance the vertical mixing across the carriage cross‐section.

### 3.2 Aerosol particle experiments

#### 3.2.1 Comparison of size fractions

**FIGURE 8:**
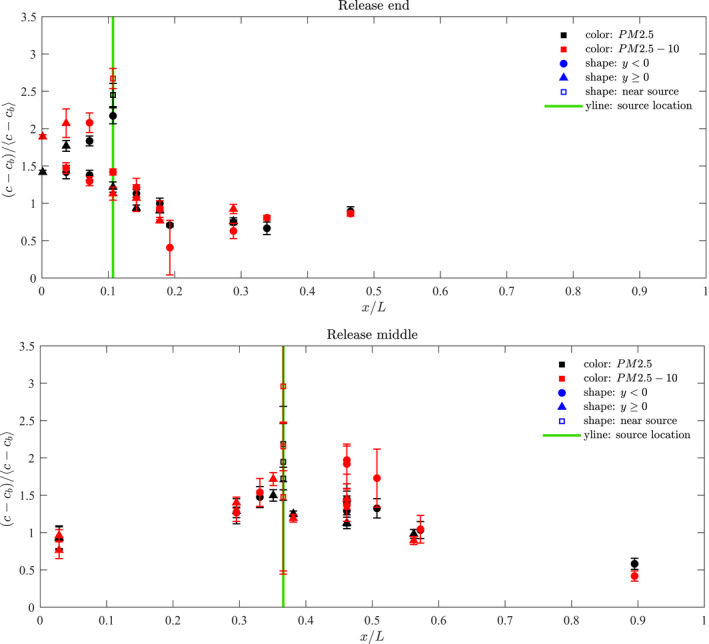
Normalised mean steady‐state concentrations of aerosol particles of both size fractions measured within carriage for a release at the end (top) and the middle (bottom). The error bars indicate the range derived from all experiments.

Figure 8 shows the normalised mean steady‐state concentrations measured within the carriage for the end release and centre release. These concentrations have been calculated by first subtracting the background concentration, $c_{b}$, then normalising by the mean steady‐state concentration for all locations. In the most part there does not seem to be a significant difference between the finer fraction, shown in red, and the coarser fraction, shown in black. However, for the end release, the coarser fraction is generally higher between the end of the carriage and the release (=−0.5<x/L<−0.4).

The similar behavior seen between the two size fractions is perhaps unsurprising given that for droplets of size range 0.1–10 μm the range in sedimentation velocity is low (10^−6^−10^−2^ ms^−1^).^19^ Typical airflow speed in buildings is around 0.1 ms^−1^, and greater velocities are expected in the carriage given the high ventilation flow rates. In this case, the airflow speed becomes the more dominant factor in determining the rate of droplet removal by deposition than the sedimentation velocities of the droplets.^19^ Using a variation of Equation 2 applied to the aerosol particle concentrations provided reasonable estimates of the fresh air intake rate, without the inclusion of an additional loss term due to deposition. We therefore expect that the deposition loss rate was small relative to the ventilation rate.

#### 3.2.2 Source at end of saloon

**FIGURE 9:**
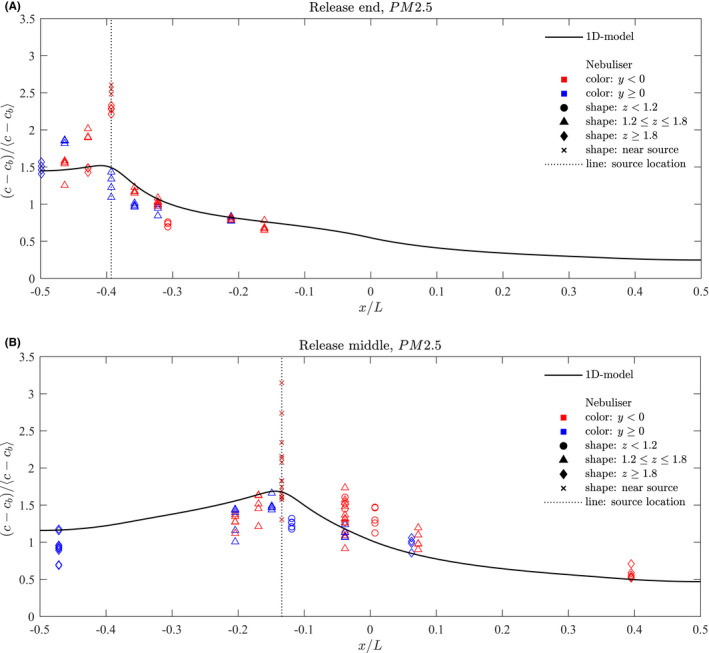
Normalised mean steady‐state concentrations of ${\mathrm{PM}}_{2.5}$ aerosol particles measured within carriage for a release at (A) the end and (B) the middle of the saloon. The full blue line shows the concentrations predicted by the 1D advection–diffusion model described in de Kreij et al.^20^

Figure 9 shows the normalised mean steady‐state of ${\mathrm{PM}}_{2.5}$ concentrations measured at each location for the experiments for which the nebuliser was located at the end of the saloon for the finer fraction. The dotted line indicates the location of the nebuliser release. The full line shows the distribution as predicted by the 1D advection–diffusion model described in de Kreij et al.,^20^ which assumes a well‐mixed cross section.

One sensor was placed at the release location itself (indicated by an x marker) and this value tends to stand out as having higher concentrations than the other locations. Another sensor was placed on the luggage rack directly above the release (indicated by a diamond marker). For the end release, this location also tended to show higher concentrations than the other locations. In this case, the release was located directly below the HVAC inlet; the suction from these inlets led to a persistent upward flow in the saloon at this location, also observed by Woodward et al.,^14^ explaining the high concentration of droplet on the luggage rack.

Concentrations remain high between the nearest end of the saloon and the source (−0.5<x/L<−0.39) but decreases gradually along the length of the carriage up to a distance of around 2 m from the source (x/L=−0.3). For distances beyond x/L=−0.3, concentrations remain fairly constant up to the centre point of the saloon (x/L=0).

Along the saloon, there are differences in concentrations between either side of the aisle (−ve and +ve y directions), however, these tend to be roughly within the scatter of points derived from the multiple experimental runs.

#### 3.2.3 Source at centre of saloon

Figure 9 also shows the normalised mean steady‐state concentrations measured at each location for the experiments for which the nebuliser was located at the centre of the saloon. As for the end experiments, the concentration measured at the source location stands out as higher than the other locations. There is also a greater variation in the values measured at this location for different experiment runs, and a steady‐state was not always reached at this location.

A large scatter is seen in concentrations between experimental runs. This could be in part due to variations in the HVAC flow rates. No clear, repeating pattern could be seen when comparing the concentrations between one side of the carriage and the other for the different runs.

A gradual decrease in concentrations occurs with distance along the carriage in both directions. For both the end and middle release, the 1D advection–diffusion model which assumes a well‐mixed cross section provides a reasonable approximation of the concentrations.

### 3.3 HVAC filter laboratory tests

Both the new and used HVAC filter samples were found to have low filter efficiencies (Equation 1), with an average of 4% across the sub‐10 micron size range of particles. The measured filter efficiency as a function of the particle size for a new and used filter is shown in Figure S5. Only a very small difference was seen in performance between the two samples. Both samples showed a greater efficiency for the smallest particles (≈0.01 μm), at nearly 20%, but this drops below 5% for particles >0.1 μm. Assuming a 4% filter efficiency, multiplying this value by the recirculation flow rates given in Table 1 gives a rate for the HVAC filters of 1.2–2.4 m^3^ min^−1^, which is 5%–8% of that of the fresh air supply rate.

Due to the porous structure of the samples, only a very small pressure drop (<10 Pa) was observed across the samples during the experiments.

Compared with face masks, these samples are not efficient in removing particles in the sub‐10 micron range. For example, typical efficiencies for medical face masks range between 74% and 88% and handmade masks 34%–60%.^2^ Further experiments are needed to evaluate their performance for particles greater than this range, however, larger particles are more likely to deposit on surfaces before reaching the HVAC inlet.

### 3.4 In‐service CO_2_ measurements

**FIGURE 10:**
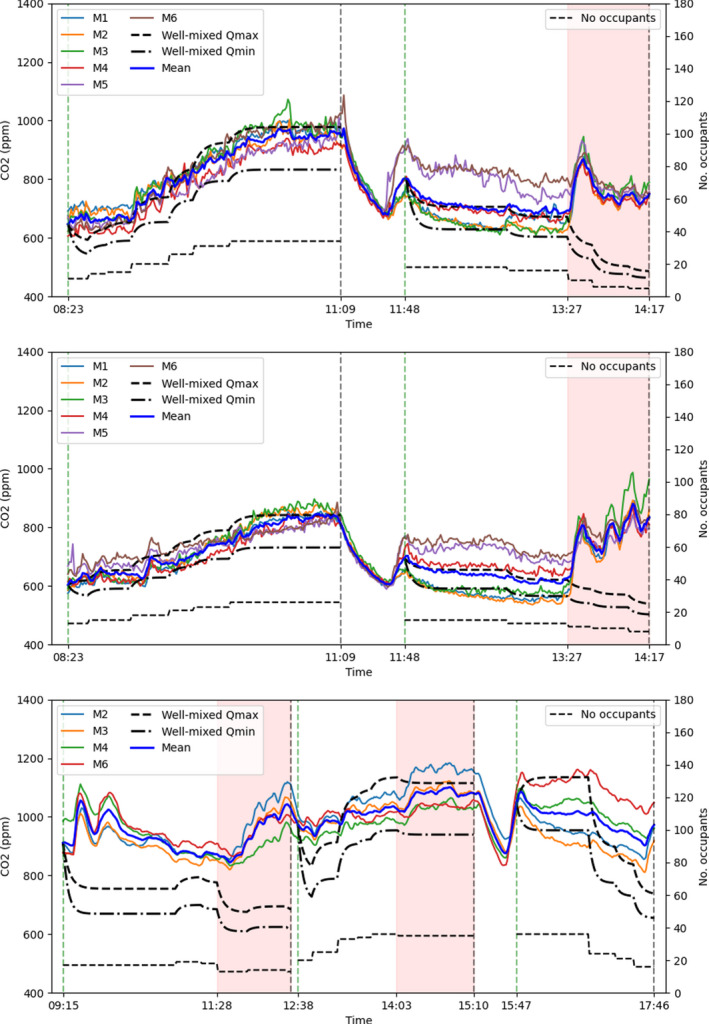
Timeseries of CO_2_ concentrations measured on the carriage during operation on the 2nd November 2020 (top), 3rd November 2020 (middle) and 23rd November 2021 (bottom). The vertical dashed lines indicate the beginning and end of the journeys and the shaded areas indicate periods during which external CO_2_ concentrations are suspected to be elevated.

The time series of CO_2_ concentrations measured by the sensors placed in the luggage racks of the carriage saloon while in service over the three separate days are shown in Figure 10. Also shown on the plots, indicated by the thin dotted line, are the number of occupants. There is a clear correlation between the number of occupants within the saloon and the measured CO_2_ concentrations. Concentrations as high as 1100 ppm are measured within the saloon during the period of greatest occupancy (35 people). Given that the seating capacity of the carriage is 88, and that the crush capacity is 160, significantly higher CO_2_ concentrations may be expected for busier services; the HVAC system for this particular carriage does not include CO_2_ monitors and does not respond to CO_2_ concentrations. These measured concentrations reinforce the concerns raised in Woodward et al.^14^ regarding the suitability of the fresh air supply rate on inter‐city carriages within the UK.

Opening the outer doors when the train has stopped does not seem to have a significant effect on the CO_2_ concentrations when measured from the luggage racks. This can be seen in Figure 10 where changes in occupancy, indicating that the train has stopped and passengers have boarded or alighted, are not accompanied by any significant decreases in CO_2_ concentrations. The train having stopped at a station, however, does not necessarily mean that the doors have opened. The external doors will only open if opened by an alighting or boarding passenger. While one of the two external doors on the carriage were likely opened at the stops, it is not clear whether only one door or both doors were open. Further, the vestibule doors close automatically after a few seconds, therefore while the external doors may have been open for a longer period of time, the vestibule doors are likely only to have been open for a few tens of seconds. For inter‐city trains such as this the time spent at stations as a proportion of total journey time tends to be low, it is therefore unlikely that air exchanged while the train is stationary has a significant effect on overall ventilation.

The shaded areas on each plot indicate periods during which increases in CO_2_ concentrations are seen due to elevated external concentrations being drawn into the carriage. For the period between 1403 and 1510 on the November 23, 2021 (lower image of Figure 10), this was due to a bonfire visible from the carriage. In each other case, the elevated CO_2_ concentrations were due to the plume of exhaust emissions from the carriage engine passing over the external HVAC inlet and resulting in the exhaust fumes being drawn into the carriage. This is known to be the source as this occurred each time the train switched from electric to diesel power. The concentrations measured during the first leg of the journey on the November 23, 2021 are suspected to be inaccurate due to an insufficient amount of time allowed for the sensors to climatise to their environment. This highlights a difficulty in using CO_2_ concentrations as a proxy for re‐breathed air and a method for evaluating the risk of airborne infection on a moving train carriage, as elevated external concentrations may lead to large over estimations of the risk within the carriage. Elevated CO_2_ concentrations were also often measured in stations, leading to elevated CO_2_ concentrations within the carriage during these periods.

Other than the excluded periods, the correlation between CO_2_ concentrations and the number of occupants suggests that these measurements provide a reasonable approximation of the concentration of re‐breathed air within the saloon. They can therefore be used to estimate the risk of airborne infection as done so in Section 3.6.

The two thick black lines in Figure 10 show the estimated CO_2_ concentrations within the saloon using the well‐mixed model given by Equation 2. The generation rate of CO_2_, G, is calculated by using an estimated CO_2_ generation rate for a typical adult at rest, taken to be 0.35 L/min^21^. Two rates of fresh air supply, Q, are assumed based on the design specification of the carriage; a low value of 20.0 m^3^/min and a high value of 26.7 m^3^/min. The exact value is not known as the carriage operated in automatic mode.

For the first two experiment days a good agreement is achieved between the well‐mixed model with the lower ventilation estimate and the mean concentrations measured within the saloon (thick blue line), with the exception of the excluded periods discussed above. For the third experiment day in 2021, the comparison is less satisfactory.

These comparisons suggest that the well‐mixed model may provide a useful method for evaluating the mean airborne risk of infection on the carriage as it is able to replicate the trend in CO_2_ concentrations in the most part. An improved comparison may be achieved with a more precise estimate of the ventilation rate on the carriage. However, it is also clear from the periods excluded from the analysis that an understanding of the incoming CO_2_ concentrations is required in order to obtain accurate estimates.

**FIGURE 11:**
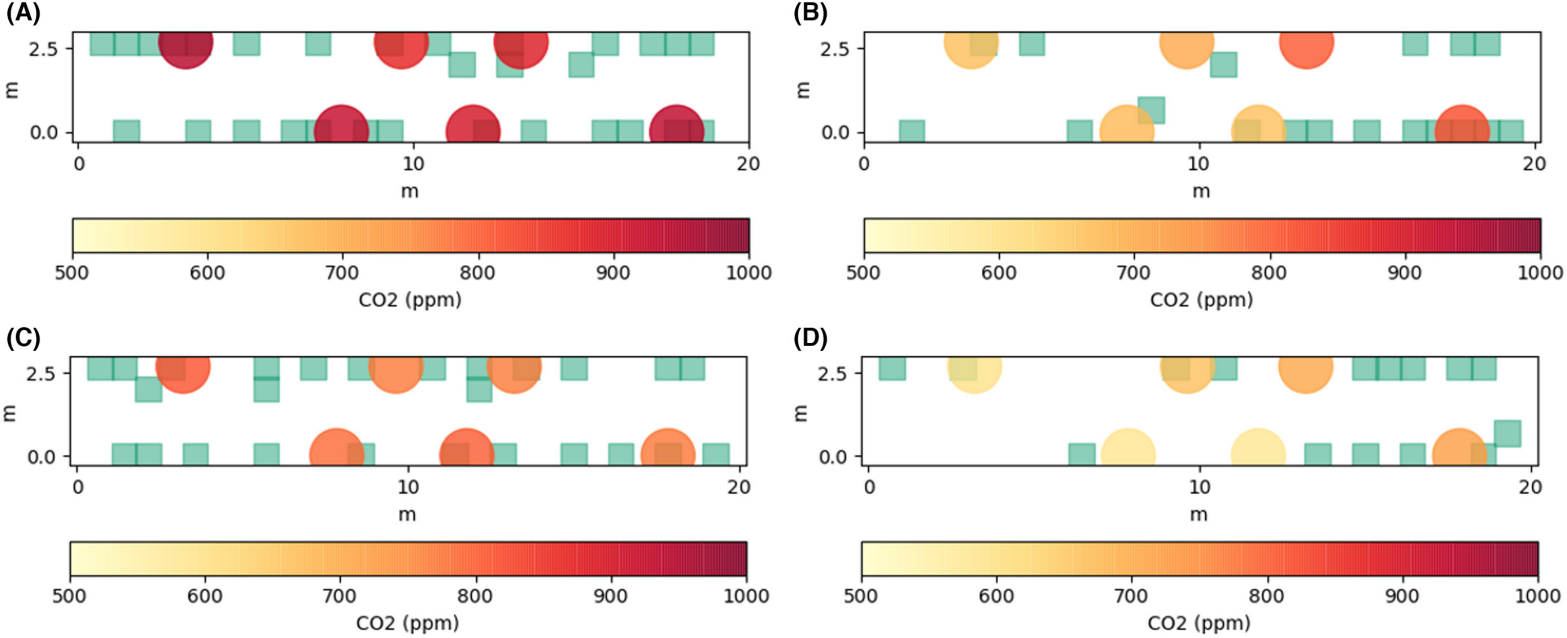
Steady‐state mean CO2 concentrations for (A) Grantham to London, 02/11, occupancy = 34, (B) London‐Grantham, 02/11, occupancy = 18, (C) Grantham‐London, 03/11, occupancy = 26, (D) London‐Grantham, 03/11, occupancy = 15. Passenger locations indicated by green squares

A significant variation in concentrations is seen within the carriage during several periods, indicating that the air within the saloon is not well‐mixed along its length as assumed by the model. Figure 11 shows the mean of the steady‐state concentrations for the London‐Grantham leg (for both journey directions) on the November 2, 2020, and passenger locations indicated by the green squares. The dependency of the CO_2_ concentrations at each sensor location on the distribution of passenger locations is clear to see from Figures 11B,D. For Figures 11A,C the passengers are fairly evenly distributed along the length of the carriage and this results in a fairly constant CO_2_ concentration along its length. A single CO_2_ monitor will therefore not always provide an accurate estimation of the concentrations throughout the carriage.

#### 3.4.1 Vertical CO_2_ profiles

**FIGURE 12:**
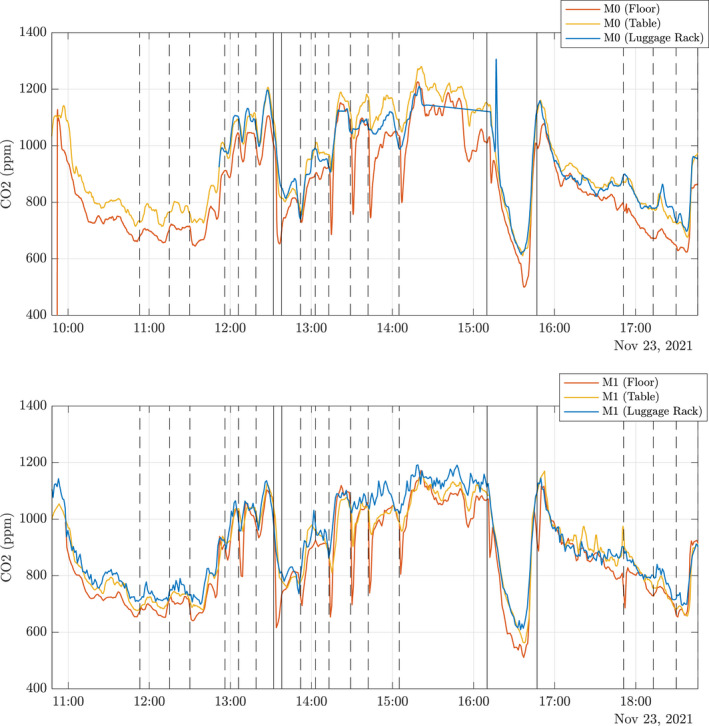
Timeseries of vertical profiles of CO_2_ concentrations measured at locations M0 (top) and M1 (bottom) in the carriage during operation on 23rd November 2021. Different colors indicate different heights of the sensors: on the floor (red), on the table, 0.7 m (yellow), on the luggage rack, 1.8 m (blue). The vertical dashed lines indicate the train entering a station, and the vertical solid lines indicate that the train is stationary in Hull or London.

Unlike the longitudinal measurements, under the HVAC inlets, the vertical CO_2_ concentrations remain well mixed throughout a journey. Figure 12 shows the CO_2_ concentrations from the six CO_2_ monitors placed at positions M0 and M1 shown in Figure 4. The monitors were placed on the floor (red), the table, 0.7 m (yellow), and in the luggage rack, 1.8 m (blue). The figure shows only small differences in vertical CO_2_ concentrations, <100 ppm comparable with the error in CO_2_ monitors (≈50 ppm). The carriage remains vertically well mixed due to the re‐circulation system in the carriage. We see the biggest variations in the CO_2_ concentrations at both the floor and table levels around stations. The reduction seen at floor level when at stations indicates that some mixing with the outdoor air is happening in stations, but it is not seen at the luggage rack height, such as in Figure 10. However, the floor level concentrations quickly return to a similar level as the two higher levels once the train has left the station. This again supports the assumption that the saloon is well‐mixed over its height. However, this also suggests that for a complete understanding of CO_2_ concentrations within the carriage, sensors placed at different vertical heights are required in addition to along the carriage length.

### 3.5 In‐service temperature measurements

During the November 2021 study, in service measurements of the vertical temperature profile were also taken. As we have seen above, although CO_2_ measurements provide good indicators for how efficient ventilation systems are at replacing old air, they are also sensitive to both outdoor concentrations and recirculating air, and do not give an indication of where exhaled breath may settle. Air exhaled through the mouth when not wearing a mask can become trapped in a layer in a thermally stratified environment if the vertical temperature gradient at breathing zone heights is sufficiently large. For temperature gradients above 0.5°C m^−1^ in an unobstructed room, air exhaled through the mouth is able to remain at breathing heights with concentrations several times the return concentration being possible.^31^

**FIGURE 13:**
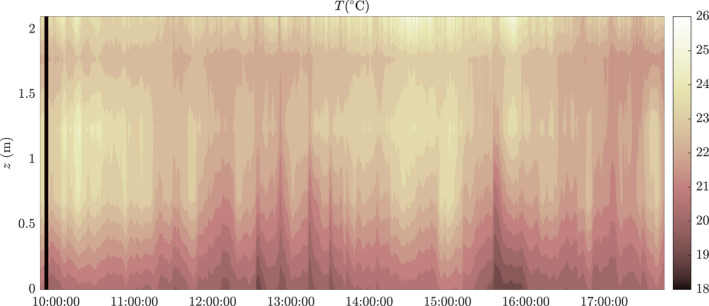
Vertical temperature profiles taken at sensor location M1 during operation on the November 23, 2021. Five sensors placed between the floor and the ceiling, contours are at 0.5°C.

Figure 13 shows a contour plot of the vertical temperature profile of the carriage during the November 2021 experiments. The sensors were placed at 5 heights in the carriage at sensor location M1: on the floor (0 m), the table (0.7 m), top of the chair (1.2 m), on the luggage rack (1.8 m), and on the ceiling (2.1 m). We observed a persistent average temperature difference between floor and ceiling of 1.6°C m^−1^ during the entire journey, with a maximum value of 3.0°C m^−1^. Also observed was a temperature inversion between the top of the chairs and the luggage rack.

The vertical temperature gradient measured here is not necessarily representative of the full carriage length. In Woodward et al.^14^ a significant difference was seen in the cross‐sectional flow pattern beneath the HVAC inlet and at the centre of the carriage. Beneath the HVAC inlet a steady upward flow was seen, with a weak downward jet from the HVAC outlet. This was in contrast to the centre of the carriage where a strong downward jet from the HVAC inlet drove the vertical mixing of air. A different temperature stratification is therefore likely at the centre of the carriage.

It is also interesting to note that the temperature stratification does not lead to a stratification in the CO_2_. In theory a “lock up layer” with elevated CO_2_, such as that observed by Bjorn & Nielsen,^31^ could exist within the carriage at a height range not picked up by the CO_2_ sensors. Our understanding of the flow structures within the carriage, and evidence of vertical mixing of CO_2_ between floor level and ceiling level suggests that this is unlikely to have occurred during our study periods. However further research is required to fully understand the implications of the strong temperature stratification observed.

### 3.6 Evaluation of risk of infection via the airborne route

Let us consider the case shown in Figure 11A for the Grantham‐London leg on the November 2, 2020, with an occupancy of 34 people. In this case, the CO_2_ concentrations are fairly constant throughout the carriage and the mean concentration is 950 ppm. Consider two higher occupancy levels of 68 and 136 and assume that the CO_2_ concentration above the outdoor concentrations, assumed to be 400 ppm, doubles as the occupancy doubles, giving concentrations of 1500 ppm and 2600 ppm, respectively. This relationship is supported by the agreement between the well‐mixed model and the measured concentrations shown in Figure 10. Further, the HVAC system of the carriage does not respond to CO_2_ concentrations, rather it is designed to maintain a certain temperature within the carriage while minimising energy consumption.

#### 3.6.1 Absolute risk estimates

Table 2 shows the probability of airborne infection during a 1 h journey for a single individual when there is I = 1 passenger infected and when there is ζ = 1/500 of the population infected, calculated using Equations 3 and 6, respectively. A value of ζ = 1/500 would indicate a low level of prevalence of the virus in the community and represents a value that may be expected when restrictions are eased, and passenger levels begin to return to pre‐pandemic levels. Assuming the lower quanta value, the risk when I=1 and when ζ=1/500 results, respectively, in a risk of 0.04% and 0.003%, for an occupancy of 34 passengers.

**TABLE 2:** Probability of infection for an individual from airborne transmission and total number of infected persons for a 1 h train journey

		I = 1		ζ = 1/500	
		q = 1 h^−1^	q = 100 h^−1^	q = 1 h^−1^	q = 100 h^−1^
n=34, T = 1 h (C = 950 ppm)	P	0.043%	4.2%	0.0028%	0.28%
	$S_{I}$	0.014	1.4	0.00093	0.091
n=68, T = 1 h (C = 1500 ppm)	P	0.043%	4.2%	0.0057%	0.56%
	$S_{I}$	0.029	2.8	0.0038	0.37
n=136, T = 1 h (C = 2600 ppm)	P	0.043%	4.2%	0.012%	1.1%
	$S_{I}$	0.058	5.6	0.016	1.5
Well ventilated office T = 8 h, Q=10l/s/p	P	0.27%	23%	–	–
Poorly ventilated office T = 8 h, Q=5l/s/p	P	0.53%	41%	–	–

*Note*: Probability of infection also calculated for a typical office building, both well ventilated and poorly ventilated, for comparison.

For I = 1, the infection risk per individual is independent of the number of occupants, as the carriage is assumed to be fully mixed. However, the number of second infections, $S_{I}$, increases, as the number of susceptible passengers increases. For ζ = 1/500, both the infection risk and the number of secondary infections are dependent on the number of occupants, as an increase of passengers leads to an increased likelihood of an infected person or persons being present. However, even at an occupancy of 136 passengers, the individual infection risk remains low at 0.01% when the lower quanta are assumed.

This risk scales close to linearly with increasing prevalence. Therefore, if we assume a higher prevalence of ζ = 1/100, the risk increases but remains low at 0.05%.

If we assume the higher, “worst‐case” quanta value of 100 h^−1^, the probability of infection to an individual per hour of travel is around 4% when assuming a single infected person is present. If we assume the near‐crush capacity of 136, the number of secondary infections is estimated to be 5.6, while this number is 1.4 for the lowest capacity of 34. If we assume a prevalence of 1/500, the greatest probability occurs for the highest occupancy case at 1%.

For the higher occupancy case, *n* = 136, assuming the lower quanta value and a prevalence of 1/500, the number of secondary infections is calculated to be 0.016 per hour of travel. Therefore, for every 64 h of near‐capacity travel, 1 new infection occurs. If we assume the higher quanta value we get 1.5 new infections per hour of travel, which equates to a new infection every 40 min of near‐capacity travel. These numbers will be considerably higher during periods of high prevalence, although it may be argued that near‐capacity services are much less likely to occur due to restrictions.

#### 3.6.2 Variation in risk within the carriage

Let us now consider the London to Grantham leg on the November 2, 2020 (Figure 11B). In this case, there is a considerable variation in CO_2_ concentrations between one end of the carriage and the other due to one side of the carriage having a higher occupancy than the other. We will make the approximation that the concentration on the left‐hand side of the carriage is 600 ppm, with an occupancy of 5, and on the right‐hand side of the carriage is 800 ppm, with an occupancy of 13. The total occupancy of the carriage is therefore 18, and the average concentration is 700 ppm. We also make an assumption that the air at either end of the saloon does not mix, that is, they are two separate volumes of air. While this is not the case in reality, given the difference in concentrations between the two ends, and the direction of bulk velocity from the centre toward either end of the saloon, it is likely to be true that passengers will be predominantly exposed to air exhaled from other passengers in the same end of the carriage.

The probabilities of infection and number of infected people on each side of the carriage are shown in Table 3. Here, we have assumed a quanta value of 1 h^−1^ and a single infected person in the carriage. Therefore, the number of infected passengers on the left‐hand side of the carriage is 5/18, and on the right‐hand side of the carriage is 13/18. The risk of infection to an individual passenger on the quieter side of the carriage is found to be 5 times lower than on the busier side.

**TABLE 3:** Probability of infection for an individual from airborne transmission and total number of infected persons for the London to Grantham leg on the November 2, 2020

	Half of carriage		Two halves combined	Full carriage
q = 1 h^−1^	n = 5, I=5/18, C = 600 ppm	n = 13, I=13/18, C = 800 ppm	n = 18, I=1, C = 600‐800 ppm	n = 18 C = 700 ppm
P	0.0081%	0.042%	0.033%	0.044%
$S_{I}$	0.00038	0.0052	0.0056	0.007

*Note*: A comparison is given between the two sides of the carriage, the average of the two sides and the full carriage assuming well‐mixed.

Taking the average risk of infection across the entire carriage gives a value of 0.033%. This is 25% lower than the risk estimated from a well‐mixed saloon assuming a concentration of 700 ppm throughout the saloon. Similarly, the number of infected passengers predicted by considering the two halves of the saloon separately is 25% lower than if the entire carriage is considered as well‐mixed.

It therefore seems that a well‐mixed assumption throughout the saloon can lead to some inaccuracy in the estimates of risk of infection and the number of secondary infections. A zonal approach such as that used in Noakes & Sleigh^32^ and Matheis et al.^11^ for a hospital may provide improved estimates. Alternatively, the one‐dimensional advection–diffusion model described in de Kreij et al.^20^ and applied to the same inter‐city carriage is able to fully resolve the longitudinal variation in risk within the carriage for different occupancies and passenger locations.

#### 3.6.3 Relative risk

While the risk estimates in Table 2 provide some idea of the expected infection risk and secondary infections, they are speculative due to the high uncertainty in the quanta estimates (see Section 2.7), and also outdated due to the quickly evolving pandemic. It is therefore also useful to consider these risk estimates relative to other typical scenarios. In Table 2, we also include estimates of the risk of infection via airborne transmission for two office scenarios. The first is a well‐ventilated office, with a fresh air supply rate of 10 liters per second per person (l s^−1^ p^−1^), which is the minimum recommended by CIBSE,^33^ and the second is an equivalent office but poorly ventilated with a fresh air supply of 5 L s^−1^ p^−1^.

Assuming a single infected person is present, the risk of infection for a day (8 h) in the well‐ventilated office is around 6 times greater than the risk from a 1‐h journey on the train. For the poorly‐ventilated office, the risk is 10–12 times greater than that for an hour long train journey, with the exact value depending on the quanta value assumed. While the risk of infection on the train is lower, this is largely due to the longer duration spent in the office, and they are not so low as to be insignificant.

While these comparisons are useful to better understand the relative risk of the train journey, it is important to understand that estimating the accumulative risk over a number of days or months to a regular commuter, and the relative contribution of a train journey as compared to time spent in the office, requires assumptions to be made regarding a number of highly uncertain factors such as the prevalence of the virus in the community, the shared journey times of each passenger and the number of repeat exposure events to an infected person. For example, repeated exposure to an infectious person over a number of days is much more likely in an office, where the same occupants return each day, than on a train, where sharing the same carriage multiple times is much less likely. People are also likely to be more active in the office and therefore generate infectious droplets at a greater rate. In contrast, the number of potential infectors is much greater for a train journey, although any time spent in the proximity of infectious people is likely to be much more brief.

## 4 CONCLUSIONS

Experiments were conducted on an inter‐city train carriage with the aim of understanding the risk of airborne infection by the SARS‐CoV‐2 virus. These experiments included in‐service CO_2_ and temperature measurements, the mapping of salt aerosol droplet concentrations released from a nebuliser and laboratory testing of the HVAC filter efficiency. These experiments were also complemented by CFD simulations which provided an understanding of the bulk airflow patterns within the saloon.

Measurements of CO_2_ can be used to estimate the risk of infection via airborne transmission, however they do not provide any insight into the different dispersion and displacement behavior for different droplet size fractions. The use of CO_2_ measurements as a proxy for re‐breathed air therefore inherently assumes that airborne droplets across the full‐size range are dispersed in the same way. For the nebuliser experiments presented here, no significant difference was seen between the dispersion of ${\mathrm{PM}}_{2.5}$ and ${\mathrm{PM}}_{2.5-10}$ droplets released within the saloon other than when released directly beneath the HVAC inlet. In this case higher concentrations were observed for the larger size fraction only within a small section of the saloon near the HVAC inlet. The similar behavior seen between the two size fractions is likely due to high airflow speeds within the carriage driven by high ventilation flow rates. When the airflow speed is of the same order or greater than the sedimentation speed of the droplets it becomes the dominant factor in determining the removal rate by deposition. Further experiments under a wider range of conditions are required to fully understand any relative differences in the dispersion of different size fractions, however, for the conditions of these experiments the results support the use of CO_2_ measurements as a proxy for re‐breathed air.

Another factor which is not captured by CO_2_ measurements is the degree of removal of infectious airborne droplets by the HVAC filters. Laboratory tests revealed that the HVAC filter material was not effective in removing droplets within the sub‐10 micron range, with an average filter efficiency across the size range of 4%. We calculated that the use of these filters provides only a 5%–8% enhancement in the removal of droplets above that provided by the provision of fresh air into the carriage.

Each of the on‐board experiments provided evidence that the saloon is not well‐mixed along its length. For the in‐service measurements, significant variation in CO_2_ concentrations were measured within the saloon when occupants were distributed unevenly. However, for the purpose of transmission risk estimates, it seems reasonable to assume that the cross‐section of the carriage is well‐mixed. This assumption is supported by the CFD simulations of the airflow and CO_2_ concentrations, the nebuliser experiments and the vertical CO_2_ measurements. It is therefore recommended that a minimum of two CO_2_ sensors are required to provide reasonable estimates of the airborne risk on the carriage. These two sensors should be placed at opposite ends of the carriage near the HVAC inlets. Further, when possible, the CO_2_ concentration of the outdoor air entering the carriage at the exterior HVAC inlet should also be monitored to avoid misleading risk estimates when external CO_2_ concentrations are higher than expected. There were several such occurrences for the three day period of in‐service measurements presented here.

The 1D advection–diffusion model described in de Kreij et al.,^20^ which assumes a well‐mixed cross‐section, was shown to compare reasonably well with aerosol concentration measurements and is therefore another useful tool for analysing the risk of infection on the carriage.

Further work is required to understand the strong vertical temperature stratification within the carriage measured directly underneath the HVAC inlet, which was not seen for the vertical CO_2_ measurements. It is unclear whether this temperature stratification extends the entire length of the carriage, or under what conditions the stratification may become stronger or weaker. Such a strong stratification raises the question of whether a “lock up layer” of breathed out air could exist, for example during periods of higher occupancy, however it seems unlikely that this occurred during the experiments presented here.

The risk of infection and number of secondary infections estimated using the CO_2_ measurements (Equation 3) are highly speculative due to the required estimate of quanta value (Section 2.7). A low and high estimate of the quanta value were therefore used to provide an upper and lower estimate. Assuming a single infected passenger is present within the carriage, the risk of infection via airborne transmission was calculated to be 0.043% for the lower quanta of 1 h^−1^. Assuming the higher quanta of 100 h^−1^, this value increased to 4.2%. This higher value is representative of a particularly infectious person or a person singing or speaking loudly. Most passengers are more likely to sit silently, or talk quietly, for the vast duration of the journey, therefore the lower estimate is likely more representative of the average case.

Given the wide range of uncertainty for these estimates, it is useful to consider the risk relative to other common scenarios. Assuming a single infected person is present, we estimated that the risk of infection for a day (8 h) in a well‐ventilated office is around 6 times greater than the risk from a 1‐h journey on the train. For a poorly‐ventilated office, the risk is 10–12 times greater than that for an hour long train journey. It is unclear to what extent such journeys increase the overall personal risk of infection for regular commuters over a number of days or weeks.

While the risk of infection for a one‐off journey is likely to be low, our analysis suggests that there is the potential for a significant number of reinfections on the carriage when a particularly infectious person is present. Every effort should therefore be made to mitigate the risk of transmission on these carriages.

## AUTHOR CONTRIBUTIONS

H.W., R.D.K., E.S.K., and P.F.L. performed conceptualisation. H.W., R.D.K., E.S.K., S.F., A.T., S.H., and S.N. performed data curation. H.W., R.D.K., E.S.K., A.T., S.H., and S.N. involved in formal analysis. P.K. and P.F.L. involved in funding acquisition. H.W., R.D.K., E.S.K., A.T., S.H., and S.N. performed investigation. M.D.W., P.K., P.F.L. collected resources and performed supervision. H.W., R.D.K., E.S.K., A.T., and S.H. performed writing—original draft preparation. H.W., S.F., S.N., A.T., S.H., and P.K. performed writing—review and editing.

## CONFLICT OF INTEREST

The authors have no conflict of interest to disclose.

## Supporting information


Appendix S1

## Data Availability

The data that support the findings of this study are available from the corresponding author upon reasonable request.
