# Supplementary material for: An evaluation of the risk of airborne transmission of COVID‐19 on an inter‐city train carriage
Source: Indoor Air. 2022 Oct 24;32(10):e13121. doi: 10.1111/ina.13121 (PMC9827851; doi:10.1111/ina.13121)
Supplement: Supplementary file 1 — Appendix S1 [file INA-32-0-s001.pdf]

1 CFD simulations setup

Unsteady Reynolds-Averaged Navier-Stokes (RANS) CFD simulations of only the carriage saloon was simulated (Figure 1); a zero transfer of air between the saloon and vestibules was assumed.

The CFD package Star-CCM+ was used with a polyhedral mesh comprising approximately 22 million cells. A mesh independence study was conducted on a segment of a carriage prior to running the simulations in order to establish a suitable mesh size. The simulations used an implicit unsteady formulation and the $k-\epsilon$ realisable turbulence model with a maximum cell size of 2 cm. The simulations were evaluated with a CFL of 10 based on the HVAC outlet velocity. The carriage walls and interior furniture were modelled as no-slip walls. For the case with passengers, the mannequins were simulated as thermal sources radiating 60W/m^2 [1, 2] with mouths having a 2cm^2 area [3] and volume flow rate of 9 l/min [4]. At this stage of modelling, where bulk transport is the focus, the average mass flow has been used as opposed to a sinusoid representing inhalation and exhalation. Furthermore, we assumed the exhaled CO_2 to have the density of air, whereas in reality CO_2 is more dense. An initial concentration of 1050ppm was assumed throughout the carriage.

The recirculation of air was modelled by dividing the inlets in two at half the length of the carriage and assuming that the flow extracted from the CFD model outlets (HVAC inlets) at one end of the carriage is recirculated exclusively in that half of the carriage. It was assumed that the CO_2 extracted from the saloon at the HVAC inlets is immediately reintroduced into the saloon via the HVAC outlet.

The concentration of CO_2 (C) entering the domain through the CFD domain inlets (HVAC outlets) is calculated as follows:

$$C_{\text{inlet}} = \frac{Q_F}{Q_F + Q_R} C_{\text{ex}} + \frac{Q_R}{Q_F + Q_R} C_{\text{outlet}} \tag{1}$$

Here C_{outlet} is the average concentration at the domain outlet (HVAC inlets) and the total flow rate from the inlets (HVAC outlets) is $Q_F + Q_R$.

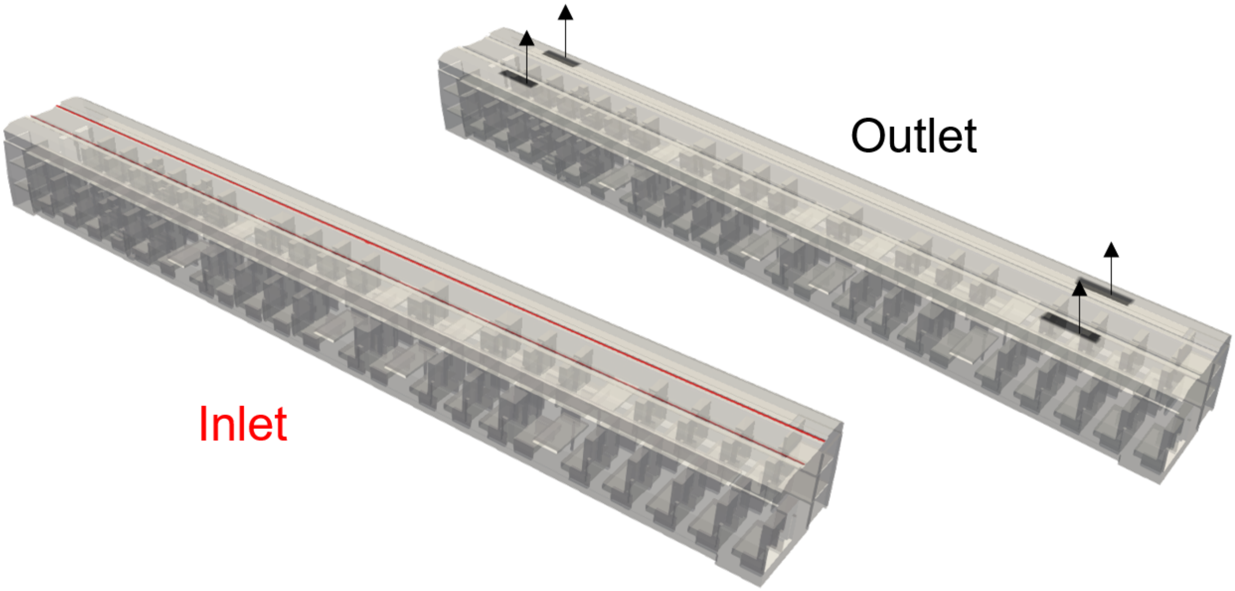

Figure 1: Train saloon geometry used for CFD simulations.

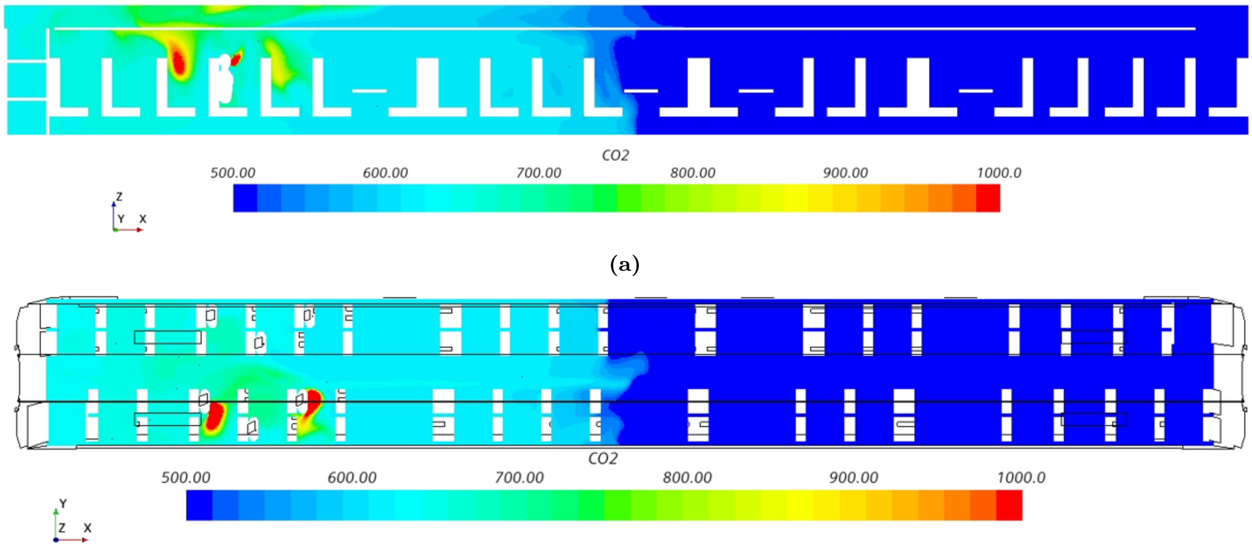

(a)

(b)

Figure 2: Plane views of CO_2 concentrations within the carriage with an occupancy of 6 passengers for (a) X-Z plane and (b) X-Y plane at Z=1.0m.

2 Filter laboratory experiments

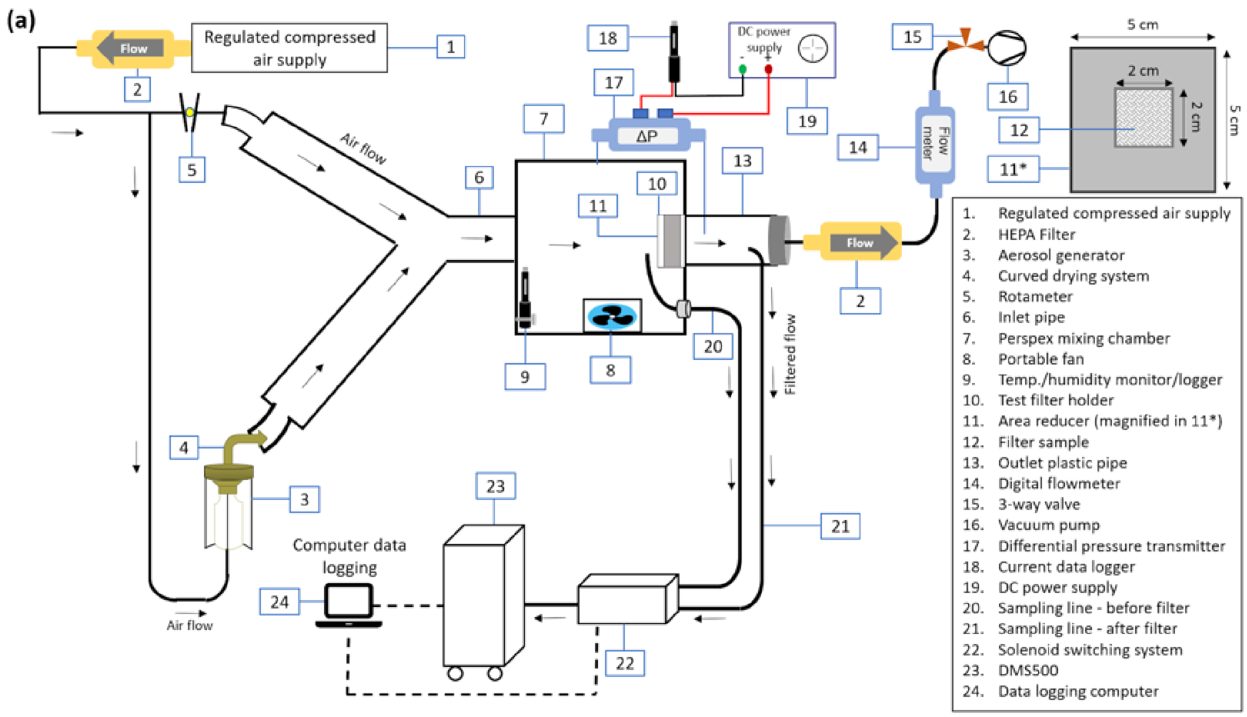

Figure 3: A schematic of the filter testing rig.

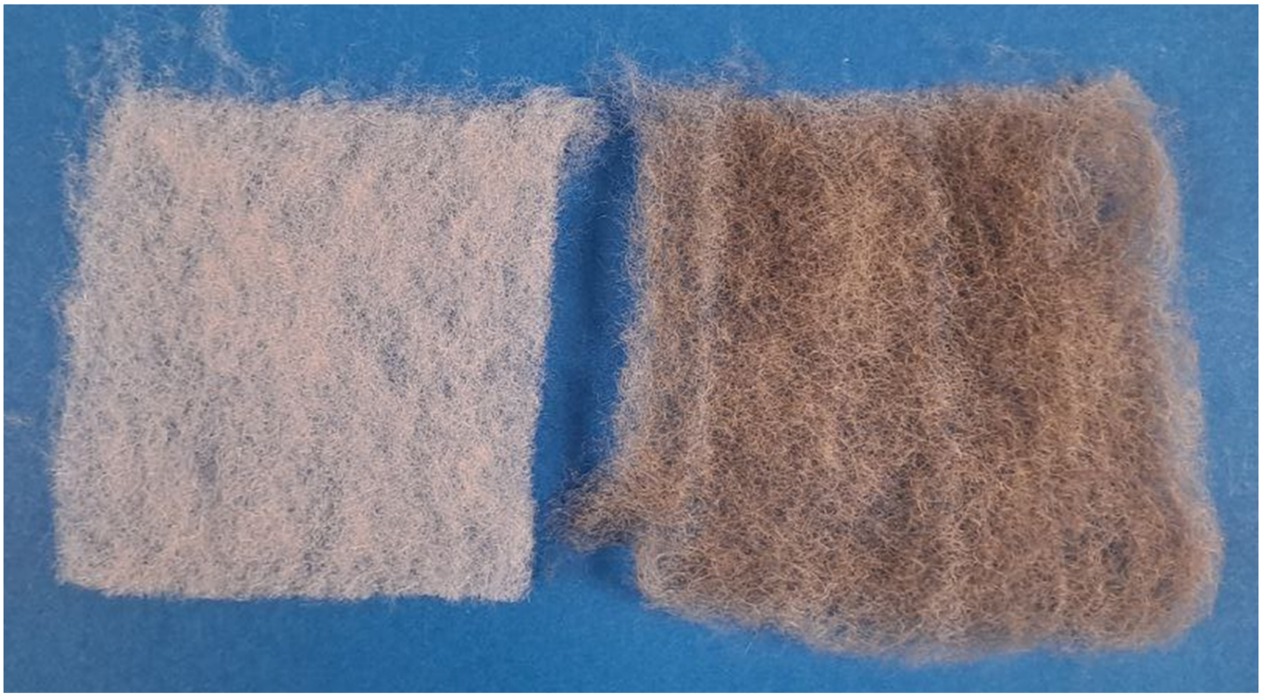

Figure 4: A new filter sample, left, and an used filter sample, right.

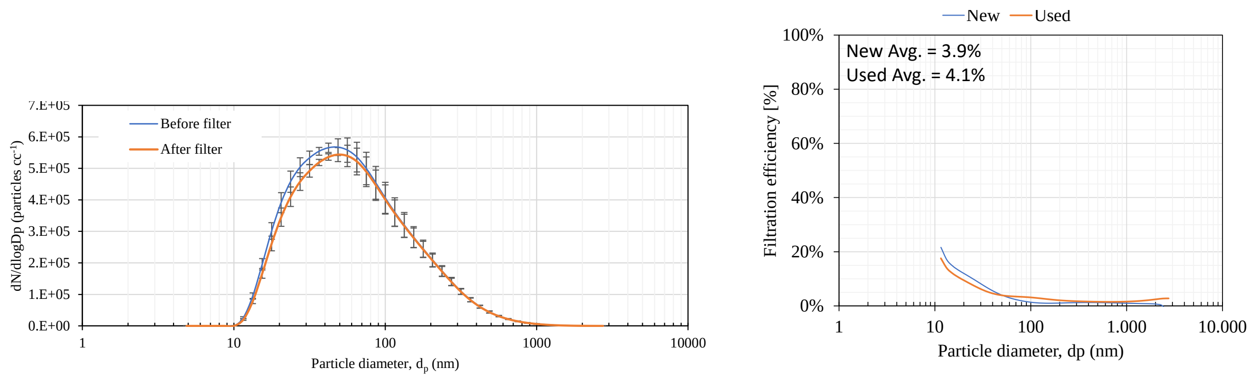

(a) Number of particles

(b) Efficiency

Figure 5: Results of a single HVAC filter test. (a) detected particles before (blue) and after (orange) filter, as function of the particle size. (b) efficiency of a new (blue) and used (orange) filter as function of the particle size.

References

[1] D Du Bois and E F Du Bois. A formula to estimate the approximate surface area if height and weight be known. *Nutrition*, 5(5):303–11, 1916.

[2] ASHRAE. ASHRAE handbook fundamentals.

[3] J K Gupta, C H Lin, and Q Chen. Characterizing exhaled airflow from breathing and talking. *Indoor Air*, 20(1):31–9, 2010.

[4] N.K. Burki. Ventilatory effects of doxapram in conscious human subjects. *Chest*, 85(5):600–604, 1984.

3. Quality control and assurance

As a part of the quality control and assurance process, we carried out a co-location study for a period of 8 hours prior to the experiments in order to assess the relative performance of all the equipment used. The correlation matrix of co-location data measured by different aerosol monitors are shown in Supplementary Information (SI) Figures S6 to S7. Further, a linear regression model was developed and applied to harmonise the raw data collected from aerosol monitors into equivalent measured data from the research grade instrument. SI Table S1 shows the coefficients of the linear regression model for each aerosol monitor with reference to the research grade instrument. A relatively high agreement was observed among all the aerosol monitors, with the Pearson correlation coefficient (r) greater than 0.85 and 0.83 for $PM_{2.5}$ and PM_{10}, respectively. Furthermore, the p-values were estimated to test the null hypothesis "correlation between aerosol monitors is zero" and were found to be insignificant (p-value <0.001).

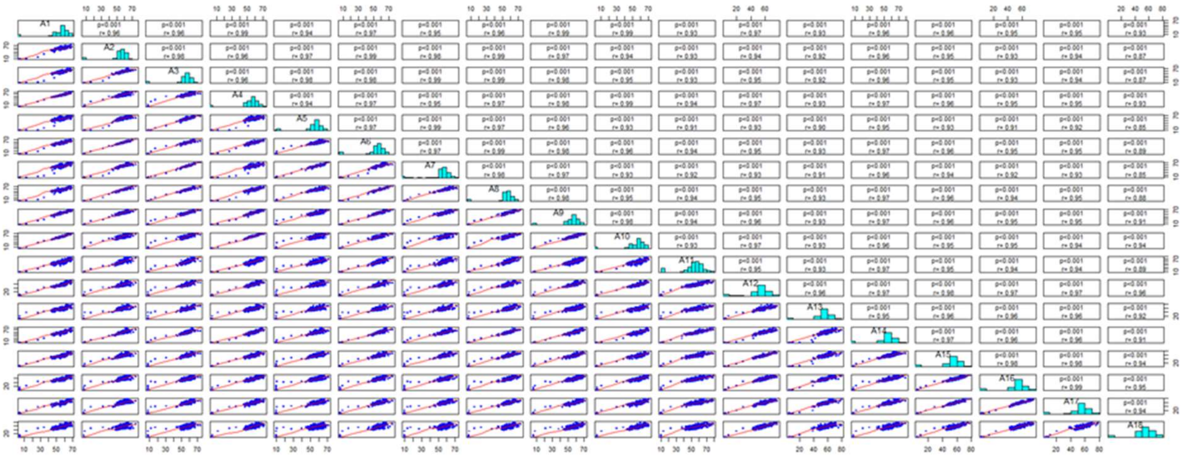

Figure S6. Correlation matrix for laser sensors (A1 to A17) put against each other and the reference aerosol monitor (GRIMM; A18) in an indoor environment with the nebuliser as a source of $PM_{2.5}$ (Pearson Correlation, *r*, is > 0.85). KCl solution at 1% concentration was used as the aerosol source in the nebuliser. The data is plotted based on the averaged 1-min time interval.

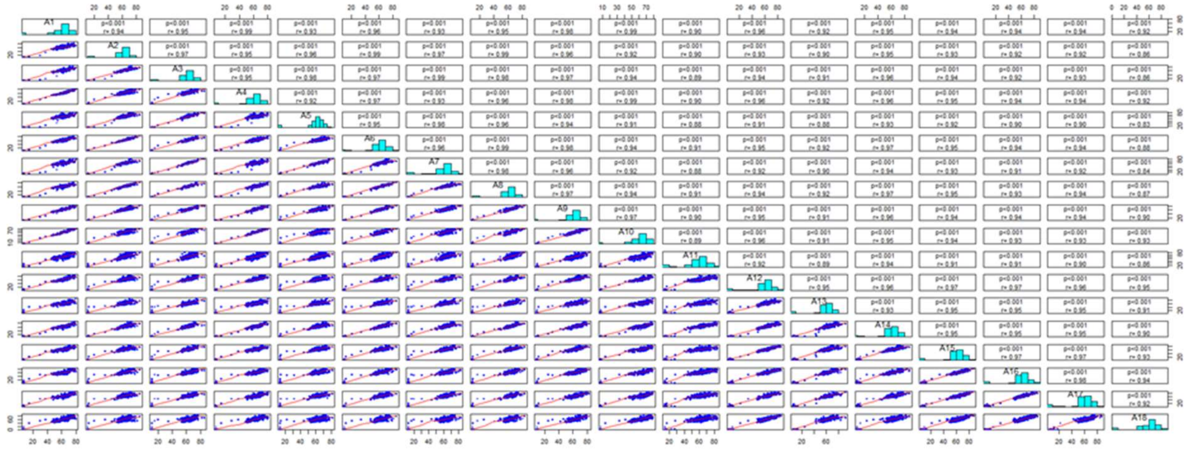

Figure S7. Correlation matrix for laser sensors (A1 to A17) put against each other and the reference aerosol monitor (GRIMM; A18) in an indoor environment with the nebuliser as a source of PM_{10} (r > 0.83). KCl solution at 1% concentration was used as the aerosol source in the nebuliser. The data is plotted based on the averaged 1-min time interval.

Table S1. The list of intercept and slope values used to convert raw data based on linear regression for $PM_{2.5}$ and PM_{10}.

Aerosol sensor	$PM_{2.5}$		PM_{10}	
	Intercept	Slope	Intercept	Slope
dy11 (A8)	6.19	0.31	9.47	0.14
dy12 (A9)	5.19	0.29	8.73	0.14
dy13 (A10)	3.57	0.26	7.19	0.11
dy3 (A1)	3.56	0.28	7.09	0.12
dy4 (A2)	6.48	0.31	10.16	0.15
dy5 (A3)	7.12	0.35	10.79	0.16
dy6 (A4)	3.40	0.33	6.49	0.17
dy7 (A5)	9.11	0.29	12.69	0.15
dy8 (A6)	5.57	0.32	9.12	0.15
dy9 (A7)	8.67	0.29	12.34	0.14
opc1 (A11)	9.99	8.74	13.90	6.33

opc2 (A12)	3.51	0.95	3.98	0.77
opc3 (A13)	4.33	1.06	4.57	0.88
opc4 (A14)	6.86	0.98	8.58	0.77
opc5 (A15)	4.44	1.12	4.63	0.92
opc6 (A16)	2.72	1.11	3.07	0.90
opc7 (A17)	4.19	1.58	5.05	1.28
